# Case Report: Asymptomatic macular edema in ozanimod

**DOI:** 10.3389/fopht.2025.1632065

**Published:** 2025-10-07

**Authors:** Valentina Annamaria Mauceri, Tommaso Torresin, Elisa Basili, Marta Gaggiola, Francesca Rinaldi, Francesco Baroni, Paolo Gallo, Paola Perini, Elisabetta Pilotto, Marco Puthenparampil

**Affiliations:** 1 Department of Neurosciences, University of Padua, Padua, Italy; 2 Ophthalmology Clinic, Azienda Ospedaliera di Padova, Padova, Italy; 3 Multiple Sclerosis Centre, Azienda Ospedaliera di Padova, Padua, Italy

**Keywords:** macular edema, ozanimod, OCT, multiple sclerosis, S1PR modulator therapy

## Abstract

We report the case of a 61-year-old patient with relapsing–remitting multiple sclerosis (RRMS) who developed asymptomatic macular edema (ME) after initiation of ozanimod, a sphingosine-1-phosphate receptor (S1PR) modulator. The patient had a history of completely resolved central serous choroidopathy (CSC) in the right eye. Following a recent clinical worsening and a new brain lesion, ozanimod was started after appropriate screening, including ophthalmological evaluation. Three months into treatment, an OCT performed as part of routine monitoring revealed ME in the contralateral (left) eye, despite the absence of visual symptoms. Ozanimod was discontinued, and ME progressively resolved over the subsequent 2 months. This case underscores the importance of ophthalmological monitoring even in asymptomatic patients, especially those with known risk factors such as prior retinal pathology. ME is a rare but recognized adverse event associated with all approved *-imod* therapies for MS, including ozanimod. Although the exact pathophysiology remains unclear, involvement of the inner blood–retina barrier via S1PR1 internalization has been hypothesized. Given ozanimod’s long half-life and active metabolites, ME resolution may be delayed after drug withdrawal. This report highlights the relevance of interdisciplinary management and the utility of OCT in early detection of asymptomatic ocular adverse events during S1PR modulator therapy.

## Introduction

Ozanimod is a sphingosine 1-phosphate (S1P) receptor 1 and 5 (S1PR_1-5_) modulator (1) approved in many countries for the treatment of relapsing–remitting multiple sclerosis (RRMS) (2–6). S1P modulators define a class of drugs that includes fingolimod, siponimod, ponesimod, and ozanimod (generically referred to as “*-imod*”) that differently bind different S1PR (7, 8). Indeed, S1P can bind five different receptors, mediating a broad range of functions in different organs, acting locally and systemically (9). The high concentration in blood is particularly relevant to maintaining the lymphocyte *trafficking*. Indeed, a lymphocyte crosses high endothelial venules thanks to a chemokine gradient (that drives the lymphocyte *homing*), but in the absence of any specific antigen recognition, they leave the lymph nodes following the S1P gradient. The relevance of the S1P gradient is further stressed by the inactivation of its receptor on lymphocytes after antigen recognition, when these cells express CD69, which blocks S1PR, hampering the egress (10). Additional evidence consists in the -*imod* induced lymphopenia that completely reverts after *-imod* discontinuation (11). The main receptor involved in lymphocyte trafficking is S1PR_1_, which is the only S1P receptor targeted by all -*imod*. Indeed, lymphopenia (from mild to life threatening) is a common, drug-class-related adverse event. In addition, the sequestration of pathogenic lymphocytes in the lymph nodes, the effect on S1PR_1_ might also be relevant for the attenuation of local inflammation driven by microglia and infiltrating T cells (12). In addition to the effect on peripheral immune system and local inflammation, S1P_1_, S1P_2_, and S1P_3_ act also on endothelial barrier, regulating their functional integrity (9, 13). Indeed, sustained concentration of fingolimod might downregulate S1PR_1_ on the surface of endothelial cells, reducing the expression of occluding, breaking the tight junctions and facilitating vascular leackage (13). This effect might explain macular edema (ME), which has been reported as an adverse event occurring in patients with RRMS treated with all S1PR. For ozanimod, the European Medical Agency states that “*Macular oedema [.] was observed with ozanimod in patients with pre-existing risk factors or comorbid conditions*,” thus recommending to perform “*ophthalmological evaluation prior to treatment initiation with ozanimod and have follow-up evaluations while receiving therapy*.” Here, we report the case of a pwMS with a previous history of completely reverted central serous choroidopathy (CSC) in the right eye who started ozanimod and developed an asymptomatic ME in the left eye that was identified by OCT after 3 months from the first administration of ozanimod and remitted progressively but slowly after 2 months. The written patient consent for data publication was obtained.

## Case description

**Figure 1:**
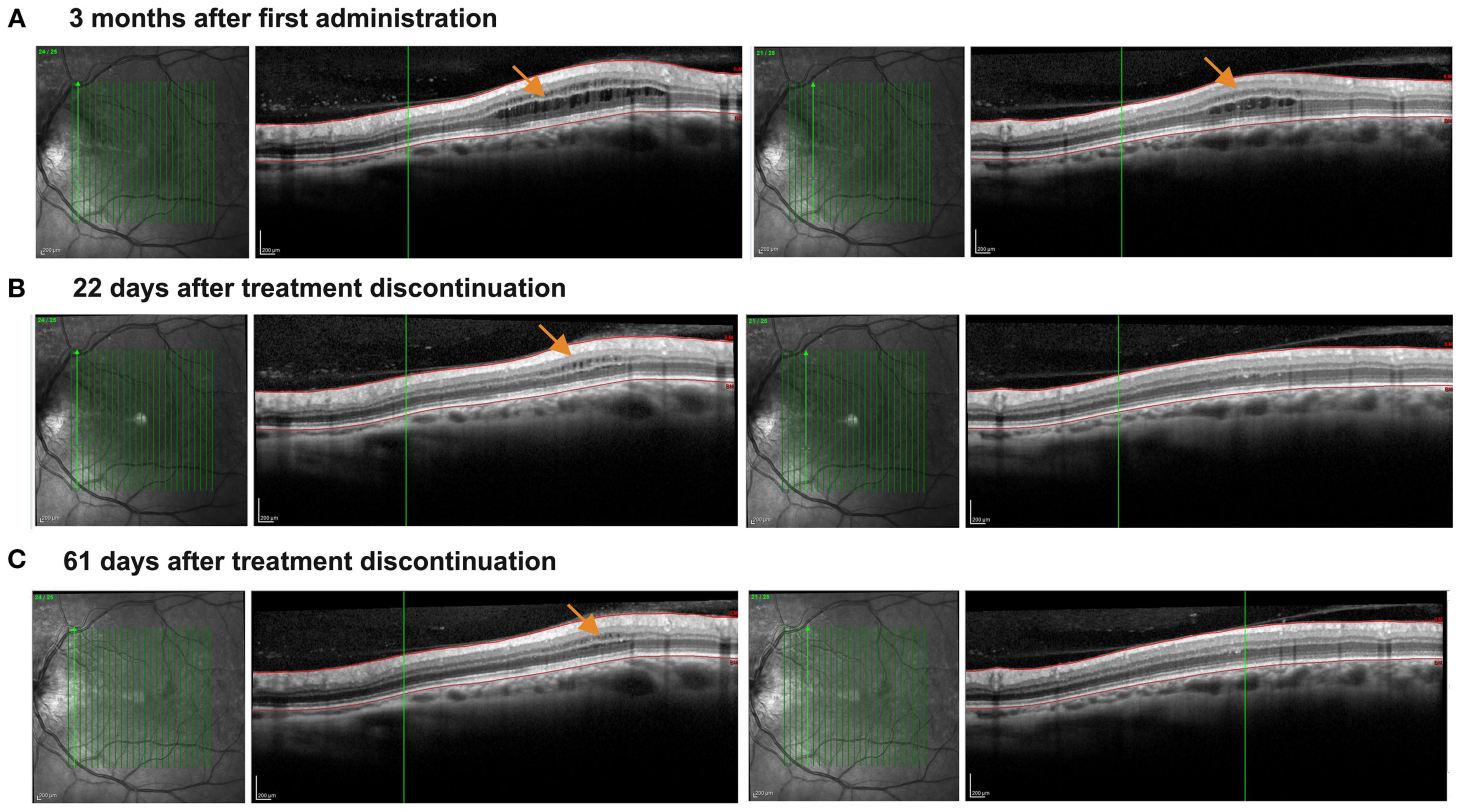
Asymptomatic development and resolution of macular edema following the treatment with ozanimod. The figure illustrates vertical macular OCT scans. **(A)** The presence of asymptomatic microcystic macular edema detected 3 months after treatment initiation (orange arrows indicate the edema), and involving both inner and our nuclear layers, which appear thickened. **(B)** Progressive reduction of the microcystic macular edema 22 days after treatment discontinuation, now involving mainly the inner nuclear layer, which is still thickened. Outer nuclear layer thickness in normal and no more microcystic macular edema is detected. **(C)** Almost complete regression of macular edema approximately 61 days after discontinuation of Ozanimod. Only a mild inner nuclear layer thickening with microcyst macular edema can be detected.

A 61-year-old patient with RRMS reported a mild but progressive reduction of left leg performance in the last 2 years. His disease onset was 20 years before, but he was clinically and radiologically stable for all these years, and he declined to start a disease-modifying treatment for MS. At the clinical evaluation performed in September 2024, a brain MRI revealed a new juxtacortical parietal lesion, while his Expanded Disability Status Scale was 3.5. Given his clinical course and the recent accumulation of a white matter lesion, a treatment with an *-imod* was indicated. Because of his age (> 61 years old), siponimod could not be administered (in Italy, if a patient’s age is higher than 61 years old, the drug is not refunded by the healthcare system), and thus ozanimod was started in November 2024, after normal blood tests (15 October 2025: Lymphocytes 1,630/µl) and dermatological, ophthalmological, and ECG evaluations. Particularly, the ophthalmological evaluation before administration was required by the medical history of CSC in the right eye. Three months later, the patient reported a subjective improvement of fatigue (that started after 500 m instead of 200 m), with a stable Expanded Disability Status Scale (3.5). While a blood examination performed at month 3 showed a moderate lymphopenia (3 February 2025: Lymphocytes 530/µl, −67.5% from baseline values in 3 months), OCT revealed asymptomatic ME in the left eye (**Figure 1A**).

### Diagnostic assessment

Due to the reported ME in ozanimod-treated RRMS, ozanimod was thus discontinued. The strong decrease of lymphocytes observed in just 3 months (about −70%) supported the strong effect of ozanimod on S1PR_1_, possibly linked to ME. During the next 61 days, OCT reported a progressive and almost complete ME resolution (**Figures 1B, C**). Moreover, the lymphocyte count also rapidly increased (26 March 2025: Lymphocytes 1200/µl), allowing us to start a different treatment for MS. Our patient started teriflunomide, discontinuing it spontaneously after a few weeks and reporting increased blood pressure values.

## Discussion

In a recent meta-analysis on randomized clinical trials, seven cases of ME (incidence rate 0.8/1,000 patient years) were reported (14). Four of them were described in pwMS treated with ozanimod 0.92 mg. All cases had pre-existing risk factors, including CSC, and developed ME 15 to 366 days after the first administration of ozanimod. In all cases but one, ozanimod was discontinued. These data confirmed that ME is a rare adverse event in ozanimod-treated pwMS. The presence of a risk factor induced us to monitor our patient, performing an OCT already 3 months after the first administration, even in the absence of any symptom. Obviously, we cannot exclude that our patient would have become symptomatic during the following days/months; actually, we believe that probably that would have been the case. Although the link between -*imod* and ME has already been described deeply from an epidemiological point of view, the mechanisms driving S1PR modulator-induced ME have not been fully clarified. Nevertheless, the action of *-imod* on S1PR_1_ expressed on the inner blood–retina barrier (iBRB) has been proposed, since the interaction with this receptor might drive its internalization, modifying the fluid homeostasis among iBRB, finally leading to intraretinal edema and foveal detachment (15). Interestingly, the S1P_R_1 is the sole receptor on which all -*imod* approved for MS act. Moreover, in all of them [Siponimod (16), Ozanimod (14), Ponesimod (17), and Fingolimod (18)], ME has been reported.

In our case ME took about 2 months to revert completely. This might be explained by the patient’s medical history but probably also by the long half-life of its principal metabolites (CC112273, 11 days). Interestingly, the almost complete elimination of ozanimod and its metabolites occurs between day 55 (5 half-lives, 97% of drug elimination) and day 66 (98.4%). In conclusion, ME is a typical adverse event in *-imod*-treated pwMS, that might also be asymptomatic but that completely reverses spontaneously. However, the long half-life of ozanimod and its metabolites might require more time from drug withdrawal for a complete resolution. In patients at risk for their medical history, a tight OCT follow-up should be planned.
